# Controlling nutritional status score predicts clinical outcome in cancer patients treated with immune checkpoint inhibitor: a systematic review and meta-analysis

**DOI:** 10.3389/fimmu.2026.1751492

**Published:** 2026-02-23

**Authors:** Lianghui Zhang, Quanli Zhang, Tao Ling, Lingli Huang

**Affiliations:** 1 Department of Oncology, Changzhou Hospital of Traditional Chinese Medicine, Changzhou, China; 2 Department of Oncology, Suqian Hospital of Traditional Chinese Medicine, Suqian, China; 3 Department of Scientific Research, Jiangsu Cancer Hospital, The Affiliated Cancer Hospital of Nanjing Medical University, Jiangsu Institute of Cancer Research, Jiangsu Key Laboratory of Innovative Cancer Diagnosis & Therapeutics, Nanjing, China; 4 Department of Pharmacy, Suqian First Hospital, Suqian, China; 5 Department of Pharmacy, Jiangsu Cancer Hospital, Jiangsu Institute of Cancer Research, The Affiliated Cancer Hospital of Nanjing Medical University, Nanjing, China

**Keywords:** cancer, controlling nutritional status, immune checkpoint inhibitor, immunonutrition, meta-analysis, prognosis

## Abstract

**Background:**

To investigate the association between pretreatment controlling nutritional status (CONUT) score and clinical outcomes for cancer patients treated with immune checkpoint inhibitors (ICIs).

**Methods:**

We conducted a comprehensive literature search of PubMed, Web of Science, Medline and Embase from inception of the databases to November 2025 to identify eligible studies concerning the relationship between pretreatment CONUT and survival outcomes in cancer patients treated with ICIs. Published data were extracted and risk ratio (RR) for objective response rate (ORR), disease control rate (DCR), and hazard ratio (HR) for overall survival (OS), progressive-free survival (PFS), along with 95% confidence intervals (CIs) were pooled. Data were analyzed using Stata14.0 software.

**Results:**

Ten studies involving 747 participants were included in this study. Patients were divided into low CONUT group and high CONUT group according to the cut-off value of CONUT score. Patients in high CONUT group had worse ORR and DCR than those in low CONUT group (RR 1.39, 95%CI 1.04-1.86;RR 1.64, 95%CI 1.32-2.03). Patients in high CONUT group had shorter PFS and OS than those in low CONUT group (1.71, 95%CI 1.21-2.42; 1.95, 95%CI 1.21-3.14). Subgroup analysis of cut-off value showed that PFS and OS of patients in high CONUT group were significantly shorter with a cut-off value of 3, and PFS of patients in high CONUT group were also worse than those of patients in the low CONUT group. Subgroup analysis of country indicated that both patients from Japan in high CONUT group had worse PFS and OS than those in low CONUT group, the OS of patients from China in high CONUT group was shorter than those in low CONUT group.

**Conclusion:**

The CONUT score has potential value as an effective biomarker for the efficacy and prognosis of cancer patients treated with ICIs. In the future, large-scale prospective cohort studies should be conducted to determine the optimal cut-off value of CONUT, and to explore whether early and proactive nutritional and anti-inflammatory support based on CONUT score can reverse the adverse prognosis.

**Systematic Review Registration:**

https://www.crd.york.ac.uk/PROSPERO/view/CRD42022378362, identifier CRD42022378362.

## Introduction

Immune checkpoint inhibitors (ICIs) have dramatically changed the cancer treatment landscape and have been widely used to treat various types of malignancies (1–3). Programmed death 1 (PD-1), programmed death ligand 1 (PD-L1) and cytotoxic T lymphocyte associated antigen 4 are three common targets. Immunotherapy has been reported to significantly improve survival in patients with a variety of cancers, including esophageal cancer, gastric cancer, lung cancer, renal cell carcinoma, hepatocellular cancer, melanoma, and more (4–7). However, ICIs are only clinically effective in specific individuals, and not all patients can benefit from them. The lack of effective prognostic markers may lead some patients to receive unnecessary ICI exposure, thereby facing significant clinical risks, most notably immune-related adverse events (irAEs). A real-world study indicates that the incidence of severe ≥ grade 3 irAEs reaches approximately 25% across various ICIs and tumor types, with the risk further increasing under combination immunotherapy (8). In addition, delayed response, hyperprogression and other phenomena of immunotherapy (9, 10), also bring difficulties and challenges to clinical treatment decision-making. Therefore, it is necessary to identify reliable prognostic markers and screen out the true beneficiaries of ICIs treatment.

Some prognostic factors were found to be related to survival of cancer patients treated with ICIs, including body mass index (11), tumor diameter (12), PD-L1 (13), tumor mutation burden (14), and tumor infiltrating lymphocytes (15). However, biomarkers based on tissue are expensive and not suitable for widespread clinical application. Malnutrition is also a common problem affecting the treatment of patients, and a number of nutritional indicators have been reported to predict cancer prognosis (16–18). In this context, the immunonutrition index, which integrates inflammatory and nutritional parameters, has attracted attention as a potential predictor of the prognosis in patients receiving immunotherapy (19). Serum albumin (ALB) is the main serum protein that reflects the nutritional status of human body, and has been shown to correlate with the prognosis of multiple tumors and can be used as a basis for risk stratification (20–22). Low level of total cholesterol (T-CHOL) was found to be associated with poorer survival and was an independent prognostic factor for cancer patients (23–25). Lymphocytopenia in cancer patients is a manifestation of impaired cellular immune function (26). Several studies have shown an association between decreased total lymphocyte count (TLC) and poor outcomes in various malignancies (27, 28). Kamath et al. (29) firstly used these three variables (ALB, T-CHOL and TLC) in a nutrition screening study involving patients from 33 hospitals in 1986. Then a new tool using these three values named controlling nutritional status (CONUT) score was developed to assess the nutritional status of patients by Ulibarri et al. (30). CONUT is simple and effective and is now used as an immuno-nutritional index (31, 32). CONUT score was calculated based on ALB, T-CHOL and TLC as follows (1). ALB ≥ 35.0, 30.0-34.9, 25.0-29.9, < 25.0 g/L were scored as 0, 2, 4, and 6 points, respectively (2). T-CHOL ≥ 180, 140-179, 100-139, < 100 mg/dl were scored as 0, 1, 2, and 3 points, respectively (3). TLC ≥ 1.60, 1.20-1.59, 0.80-1.19, < 0.80 × 109/L were scored as 0, 1, 2, and 3 points, respectively. CONUT score was divided into four grades: normal (0 to 1), mild (2 to 4), moderate (5 to 8), and severe (9 to 12) (33, 34).

CONUT score has been reported to predict the prognosis of patients with various cancers, including gastric cancer, colon cancer, and pancreatic cancer (35–37). CONUT assessed before chemotherapy was found to be an independent prognostic factor for overall survival (OS) in patients with advanced pancreatic cancer receiving a multiagent chemotherapy regimen (38). For metastastic colorectal cancer patients treated with first-line chemotherapy, progression-free survival (PFS) and OS were significantly shorter in the group with higher CONUT scores (39). Shimose et al. evaluated the prognostic factors of OS in 164 patients with hepatocellular carcinoma treated with lenvatinib and showed that CONUT score was an independent factor for OS (40). A number of related studies have explored this in recent years. In non-small cell lung cancer patients treated with pembrolizumab, patients with poor nutritional status (CONUT score of ≥ 3) had significantly shorter PFS and OS compared with patients with good nutritional status (41). Takemura et al. assessed the CONUT score of 43 patients with advanced renal cell carcinoma treated with nivolumab and reported that a CONUT score of 5 or more was independently associated with poor PFS (42). Due to the small number of studies and single-center studies, the effect of the CONUT score on the prognosis of cancer patients treated with ICIs is currently unclear.

In this study, we systematically reviewed publications on the relationship between CONUT and the prognosis of cancers patients of any stage and performed this meta-analysis to demonstrate the prognostic effect of pretreatment CONUT on treatment response, PFS, and OS in cancer patients treated with ICIs.

## Methods

This study was performed following the Preferred Reporting Items for Systematic Reviews and Meta-Analyses (PRISMA) guidelines (43). This study protocol was registered in PROSPERO (No. CRD42022378362, https://www.crd.york.ac.uk/PROSPERO/view/CRD42022378362).

### Literature search

A comprehensive search for English language studies was conducted in PubMed (NLM), Web of Science (Clarivate), Medline (Clarivate) and Embase (Elsevier platforms) from inception of the databases to November 2025 (L Zhang, T Ling and L Huang). The following key words were used: (“controlling nutritional status” OR “CONUT”) AND (“neoplasms” OR “carcinoma”) AND (“immune checkpoint inhibitors” OR “PD-L1 inhibitor” OR “PD-1 inhibitor”). Full database search strategies can be viewed in the **Supplementary Data Sheet 1**.

### Study selection

The inclusion criteria were as follows: 1) studies were accessible in English; 2) studies including cancer patients treated with ICIs; 3) available data for calculating survival estimates, including hazard ratio (HR) or risk ratio (RR) with 95% confidence intervals (CIs); 4) CONUT were calculated before ICIs treatment; 5) cut-off value of pretreatment CONUT was obtainable. Reviews, case reports, letters and editorials were excluded. If trials had multiple publications, the latest and most complete report was adopted. Three reviewers (L Zhang, T Ling and L Huang) independently screened title/abstract and reviewed full text.

### Patient and public involvement

No patient was involved.

### Data extraction and quality assessment

Two reviewers (T Ling and L Huang) independently extracted data from the included studies using a pre-designed excel data extraction form, including the following variables: study, country, cancer type, sample, the sample of low/high CONUT, study design, gender, age, immunotherapy drug, cut-off value, CONUT score, follow-up time and endpoint. RRs with 95%CIs for objective response rate (ORR), and disease control rate (DCR) were also extracted if available. Both HRs or RRs with 95%CIs in univariate and multivariable analyses were extracted for analysis.

The Newcastle-Ottawa Scale (NOS, scores of 0–9 stars) was applied to assess the quality of enrolled publications (44). An article with NOS score ≥ 7 was regarded as a high-quality study. Two reviewers (T Ling and L Huang) assessed each study independently and reached a consensus after discussion.

### Statistical analysis

The primary outcomes were OS and PFS, expressed as pooled HRs with 95%CIs. OS was defined as the time from the first day of ICIs treatment to the date of death or last follow-up, and PFS was defined as the time from the first day of ICIs treatment to the date of documentation of disease progression, death, or last follow-up. In this study, all follow-up periods from each study and data from both uni- and multivariable analyses were entered to enable sufficient data for these analyses. When the same study had undertaken both univariable and multivariable analyses, only data from the multivariable analysis were included. This meta-analysis prioritizes the inclusion of original studies that employ multivariate analyses to control for potential confounding factors, thereby reducing the impact of confounding bias on the effect size. The secondary outcomes were ORR and DCR, expressed as pooled RRs with 95%CIs. ORR was defined as the proportion of patients who achieved complete or partial response, and DCR was defined as the proportion of patients who achieved complete response, partial response, or stable disease according to the RECIST criteria. Estimates were first summarized using the fixed-effects model. If heterogeneity was significant, the random-effects model was applied. The Chi-square test and I^2^ statistic were used to assess the statistical heterogeneity among articles. *P* < 0.05 being considered statistically significant or I^2^ > 50% indicating higher heterogeneity. Subgroup analysis was used to analyze the sources of heterogeneity. A sensitivity analysis was performed using the leave-one-out sensitivity method to evaluate the robustness of the combined results and to identify the studies that contributed significantly to heterogeneity. A graphical funnel plot and Egger’s test was used to evaluate publication bias. All tests were two-tailed and a P-value less than 0.05 was considered statistically significant. Statistical analyses were conducted by using Stata 14.0 (Stata Corporation, College Station, TX, United States).

## Results

### Literature search

**Figure 1:**
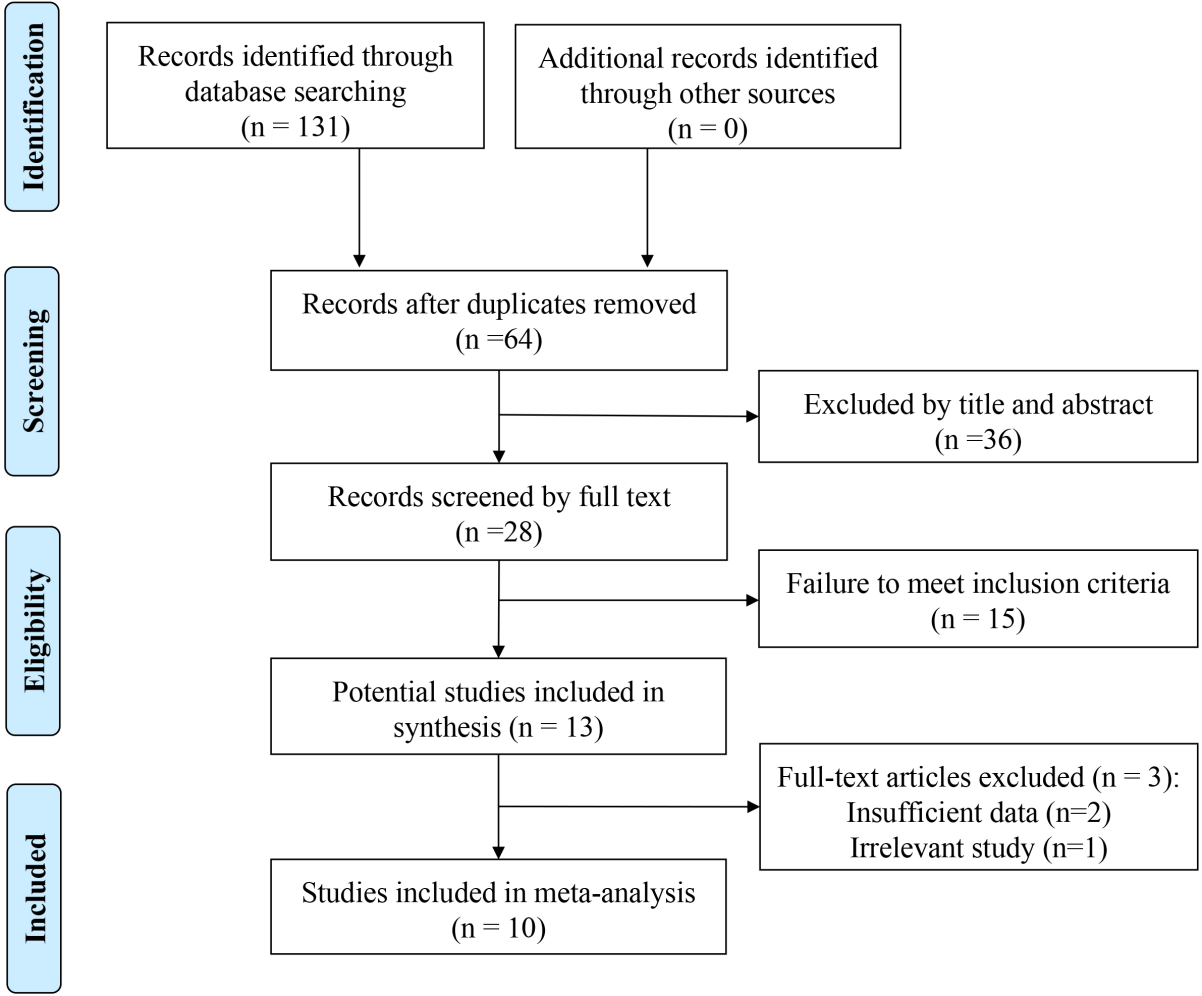
Flow diagram for the identification of included studies.

The flowchart of literature search illustrates the selection process (**Figure 1**). Database searching identified 130 records with a total of 64 duplicates identified and omitted. Title abstract screening was performed on 64 records and 15 records went through full text review. After excluding 3 papers due to failure to meet inclusion criteria, ten articles (33, 34, 41, 42, 45–50) involving 747 patients were included in this meta-analysis.

### Study characteristics and quality assessment

The main characteristics of the eligible articles are depicted in **Table 1** and the outcome measures of included studies are depicted in **Supplementary Table S1**. All included studies were retrospective research. Four of the ten studies were from Japan and six (33, 34, 45, 47, 48, 50) were from China. The determination of high or low CONUT depends on the cut-off value, and different articles have different cut-off values. In the studies we included, CONUT cut-off values ranged from 1 to 4 across studies with 1 (34), 2 (42, 45, 48, 50), 3 (33, 41, 49) and 4 (46, 47). All of these studies reported HRs for PFS, and eight (34, 41, 45–50) of them reported HRs for OS. Four studies (41, 45, 46, 49) reported ORR and DCR. The assessment of study quality for all included trials is summarized in **Supplementary Table S2**. The NOS scores of ten included articles were ≥ 8, indicating high quality.

**Table 1:** The characteristics of included studies.

Study	Country	Cancer type	Sample	Low/high CONUT	Study design	Gender (M/F)	Age (year), median (range)	Immunotherapy drug	Cut-off value	CONUT score, median (range)	Follow-up time (month)	Endpoint
Taro Ohba 2019 (41)	Japan	Stage III or IV NSCLC	32	22/10	Retrospective	29/3	65 (44–85)	Pembrolizumab	3	2.59 (0–9)	12	ORR, DCR, PFS, OS
Kosuke Takemura 2020 (42)	Japan	Stage IV RCC	49	24/25	Retrospective	37/12	NR	Nivolumab	2	2 (0-4)	26.4 (7.2-33.8)	PFS
Lele Chang 2022 (45)	China	Progression or recurrence after neoadjuvant or adjuvant therapy or advanced EC	69	42/27	Retrospective	67/2	60 (44-78)	Camrelizumab or sintilimab or toripalimab	2	1.62 (0-7)	18.3	ORR, DCR, PFS, OS
Li Chen 2022 (34)	China	Stage I or II or III or IV GC	146	75/71	Retrospective	102/44	59 (34-82)	NR	1	NR	NR	PFS, OS
Xiaofeng Chen 2022 (33)	China	Advanced HCC	20	12/8	Retrospective	18/2	56 (41-70)	Sintilimab	3	1.5 (0-8)	14.7(6.6-21.9)	PFS
Akihiro Sakai 2023 (46)	Japan	Recurrent or metastatic head and neck squamous cell carcinoma	51	34/17	Retrospective	48/3	66 (47-83)	Nivolumab or Pembrolizumab	4	NR	NR	ORR, DCR, PFS, OS
Xiao-Han Zhao 2023 (47)	China	Recurrent or metastatic esophageal squamous cell carcinoma	48	30/18	Retrospective	32/16	65	Camrelizumab	4	3.31 (0-6)	NR	PFS, OS
Zhengfeng Zhang 2023 (48)	China	Advanced biliary tract cancer	129	59/70	Retrospective	81/48	60.12	Camrelizumab, pembrolizumab, toripalimab and others	2	NR	NR	PFS, OS
Ken Horisaki 2025 (49)	Japan	Metastatic malignant melanoma	123	67/56	Retrospective	68/55	67.0 (28-87)	Nivolumab, pembrolizumab, or nivolumab plus ipilimumab	3	NR	NR	ORR, DCR, PFS, OS
Yu-Xuan Zhu 2025 (50)	China	Advanced non-small cell lung cancer	80	32/48	Retrospective	53/27	66.79 (39-84)	Pembrolizumab, atezolizumab, or tislelizumab	2	2.28 (0–6)	NR	PFS, OS

NSCLC, non-small cell lung cancer; RCC, renal cell carcinoma; EC, esophageal cancer; GC, gastric cancer; HCC, hepatocellular carcinoma; PFS, progression-free survival; OS, overall survival; ORR, objective response rate; DCR, disease control rate; NR, not reported.

### Association between CONUT and ICIs treatment response

**Figure 2:**
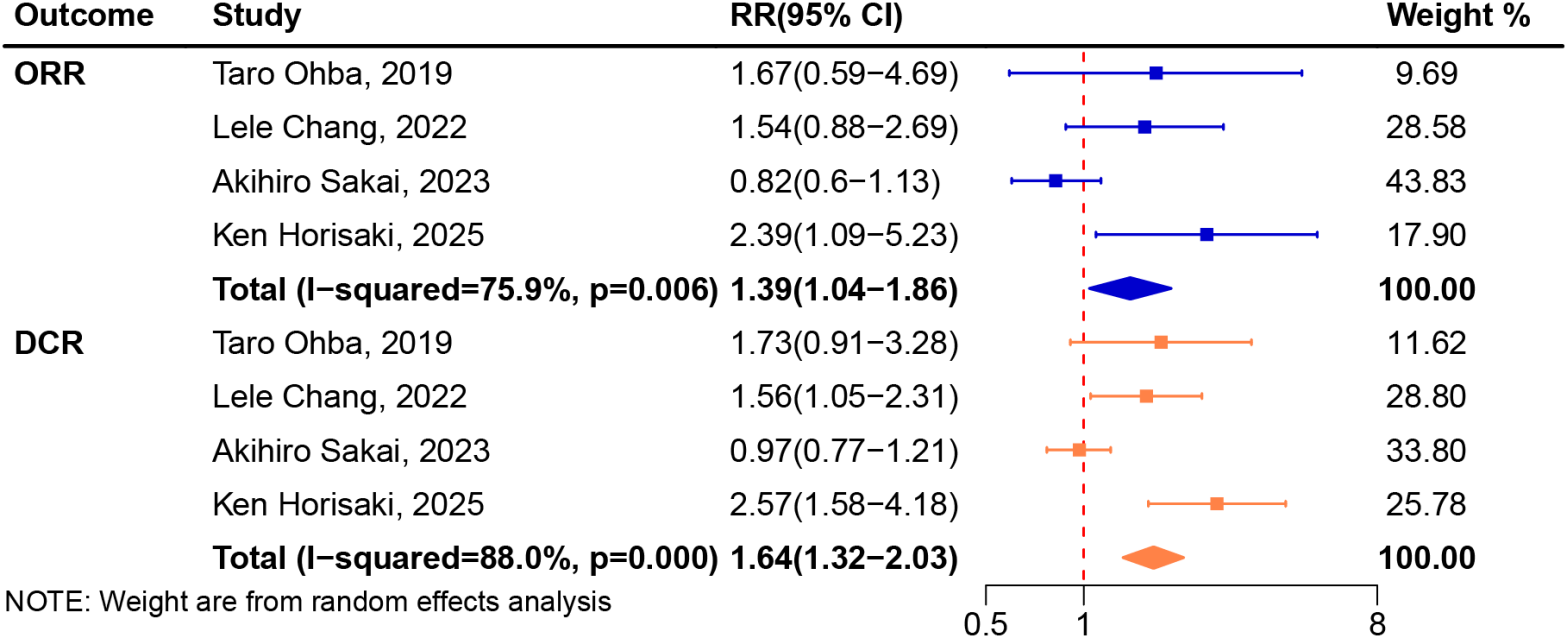
Meta-analysis of impact of CONUT on objective response rate and disease control rate treated with immune checkpoint inhibitors.

A total of four trials (41, 45, 46, 49) involving 275 patients were included in the analysis. We applied the random-effects model for pooled analysis of RR of ORR with high heterogeneity (I^2^ = 75.9%, *P* = 0.006). There was significant difference in ORR between high CONUT group and low CONUT group (RR 1.39, 95%CI 1.04-1.86, **Figure 2**).

A total of four trials (41, 45, 46, 49) involving 275 patients were included in the analysis. We applied the random-effects model for pooled analysis of RR of DCR with high heterogeneity (I^2^ = 88.0%, *P* < 0.001). Pooled analysis revealed that patients treated with ICIs in low CONUT group had better DCR compared with high CONUT group (RR 1.64, 95%CI 1.32-2.03, **Figure 2**).

### Association between CONUT and survival

**Figure 3:**
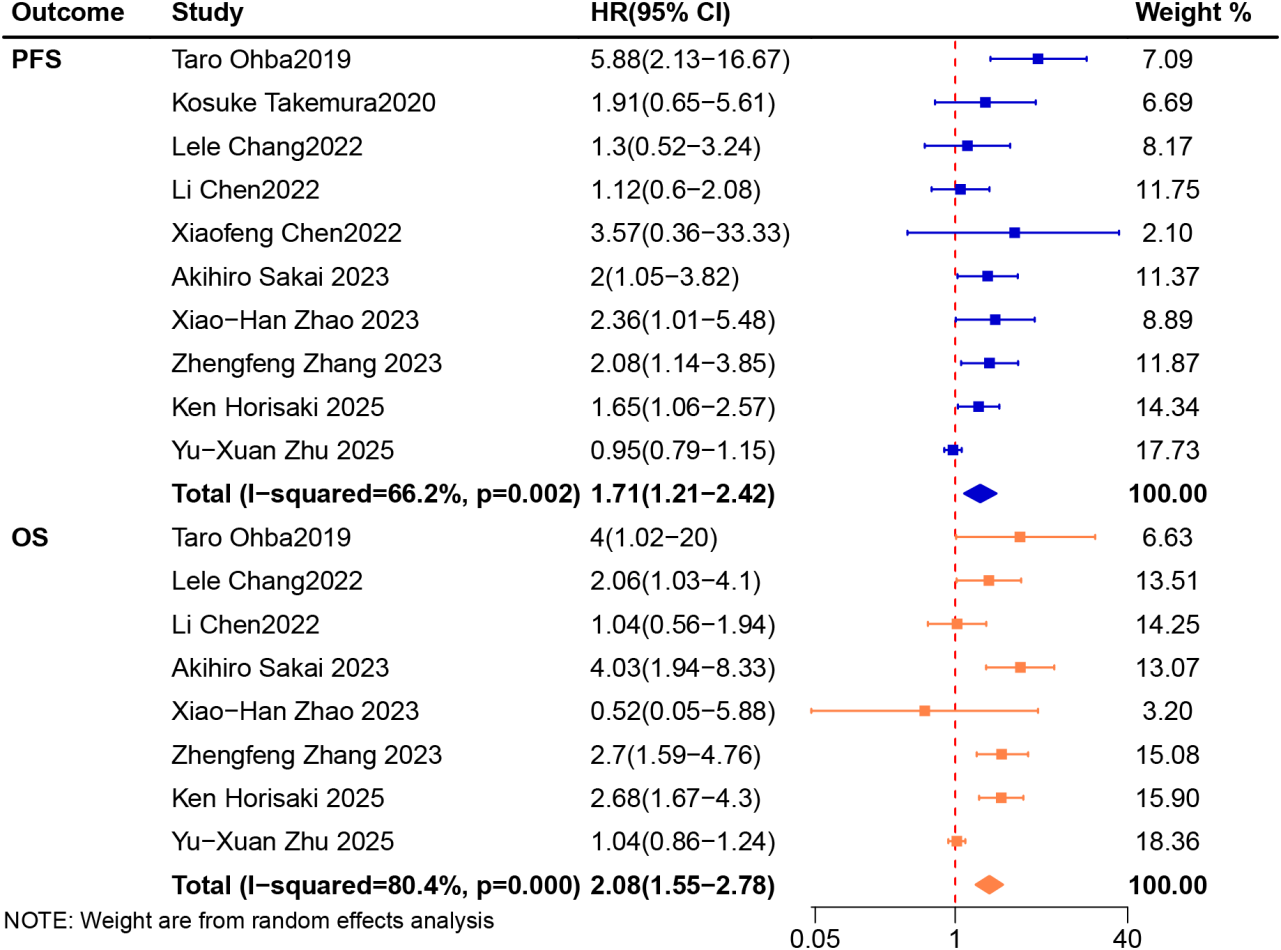
Meta-analysis of impact of CONUT on progressive-free survival and overall survival of patients treated with immune checkpoint inhibitors.

A total of ten trials (33, 34, 41, 42, 45–50) involved 747 patients and provided PFS data were included in the analysis. We applied the random-effects model for pooled analysis of HRs of PFS with high heterogeneity (I^2^ = 66.2%, *P* = 0.002). The results showed that patients receiving ICIs in the low CONUT group had a longer PFS than those in the high CONUT group (HR 1.71, 95%CI 1.21-2.42, **Figure 3**). Subgroup analysis of cut-off value showed that the PFS of patients with CONUT ≥ 3 was significantly shorter than that of patients with CONUT < 3 (HR 2.86, 95%CI 1.08-7.58) and the PFS of patients with CONUT ≥ 4 was also significantly shorter than that of patients with CONUT < 4 (HR 2.12, 95%CI 1.27-3.55). No difference was found in PFS between patients with CONUT ≥ 2 and CONUT < 2. Subgroup analysis of country showed that both patients from Japan and China in high CONUT group had worse PFS than those in low CONUT group (HR 2.00, 95%CI 1.44-2.78; HR 1.65, 95%CI 1.16-2.34). In cut-off value of 4, Japan and China subgroups, the heterogeneity was significantly decreased. The results of the specific subgroup analysis are presented in **Table 2**.

**Table 2:** Subgroup analysis of CONUT on progression-free survival and overall survival.

Outcome	Subgroup	Subgroup Category	No. of Included Studies (n)	Sample Size (n)	RR/HR (95%CI)	Heterogeneity Test
PFS	Cut-off value	Cut-off value of 2	4	327	1.36 (0.84, 2.19)	58.6% (*P* = 0.064)
		Cut-off value of 3	3	175	2.86 (1.08, 7.58)	61.4%(*P* = 0.075)
		Cut-off value of 4	2	99	2.12 (1.27, 3.55)	0.0% (*P* = 0.763)
	Country	Japan	4	255	2.00 (1.44, 2.78)	39.4% (*P* = 0.175)
		China	5	412	1.65 (1.16, 2.34)	0.0% (*P* = 0.485)
OS	Cut-off value	Cut-off value of 2	3	278	1.72 (0.86, 3.41)	84.8% (*P* = 0.001)
		Cut-off value of 3	2	155	2.78 (1.77, 4.36)	0.0% (*P* = 0.615)
		Cut-off value of 4	2	99	2.04 (0.31, 13.51)	60.1% (*P* = 0.113)
	Country	Japan	3	206	3.08 (2.10, 4.52)	0.0% (*P* = 0.614)
		China	4	392	1.71 (0.98, 2.98)	51.7% (*P* = 0.102)

PFS, progression-free survival; OS, overall survival; HR, hazard ratio; RR, risk ratio; CI, confidence interval.

A total of eight trials (34, 41, 45–50) which involved 678 patients and provided OS data were included in the analysis. We applied the fixed-effects model for pooled analysis of HRs of OS with high heterogeneity (I^2^ = 80.4%, *P* < 0.001). The combined analysis showed that high CONUT group had worse OS than low CONUT group (HR 2.08, 95%CI 1.55-2.78, **Figure 3**). Subgroup analysis of cut-off value showed that the OS of patients with CONUT ≥ 3 was significantly shorter than that of patients with CONUT < 3 (HR 2.78, 95%CI 1.77-4.36). There were no significant differences in OS between the two groups when the cut-off values were 2 (HR 1.72, 95%CI 0.86-3.41) and 4 (HR 2.04, 95%CI 0.31-13.51). Subgroup analysis of country showed that the OS of patients from Japan in high CONUT group was shorter than that of patients in low CONUT group (HR 3.08, 95%CI 2.10-4.52). There was no statistical difference in OS between patients with high CONUT group and low CONUT group from China (HR 1.71, 95%CI 0.98-2.98). In cut-off value of 3 and Japan subgroups, the heterogeneity was significantly decreased. The results of the specific subgroup analysis are presented in **Table 2**.

**Figure 4:**
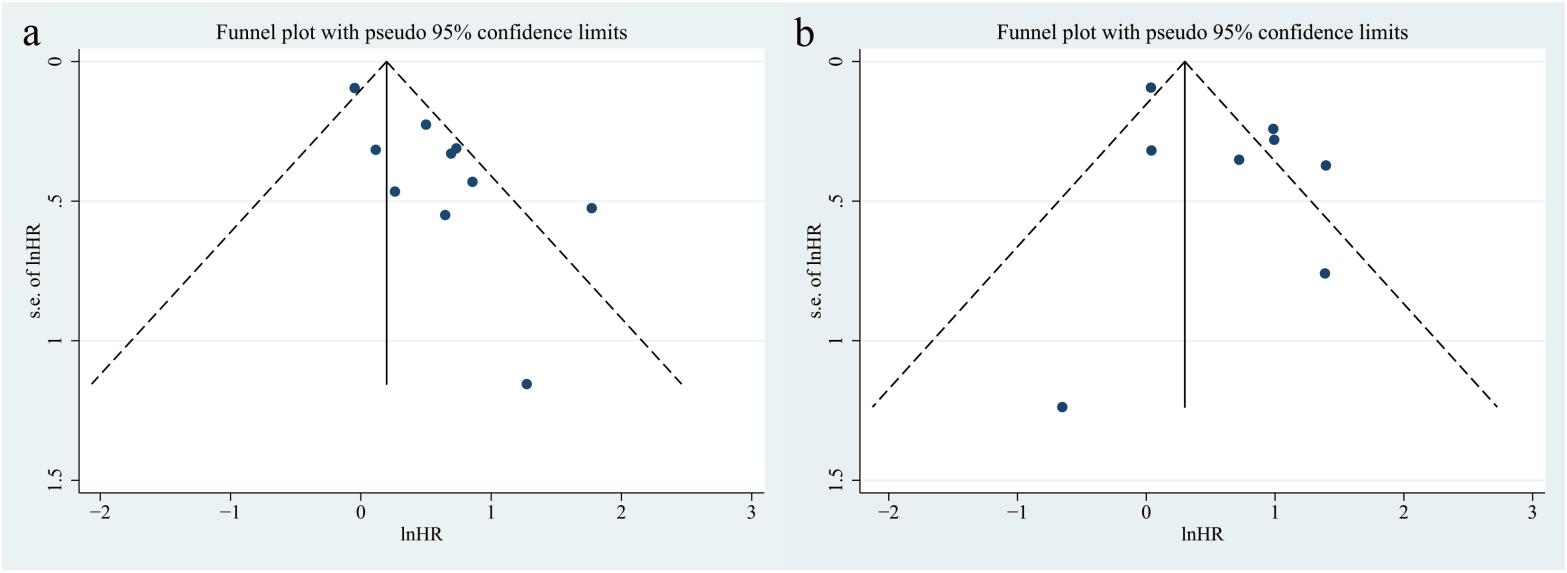
Funnel plots of CONUT on progressive-free survival **(a)** and overall survival **(b)** in patients treated with immune checkpoint inhibitors.

The funnel plots for CONUT in OS and PFS were symmetrical, indicating no publication bias (PFS, P = 0.131; OS, P = 0.816; **Figure 4**).

### Sensitivity analysis

**Figure 5:**
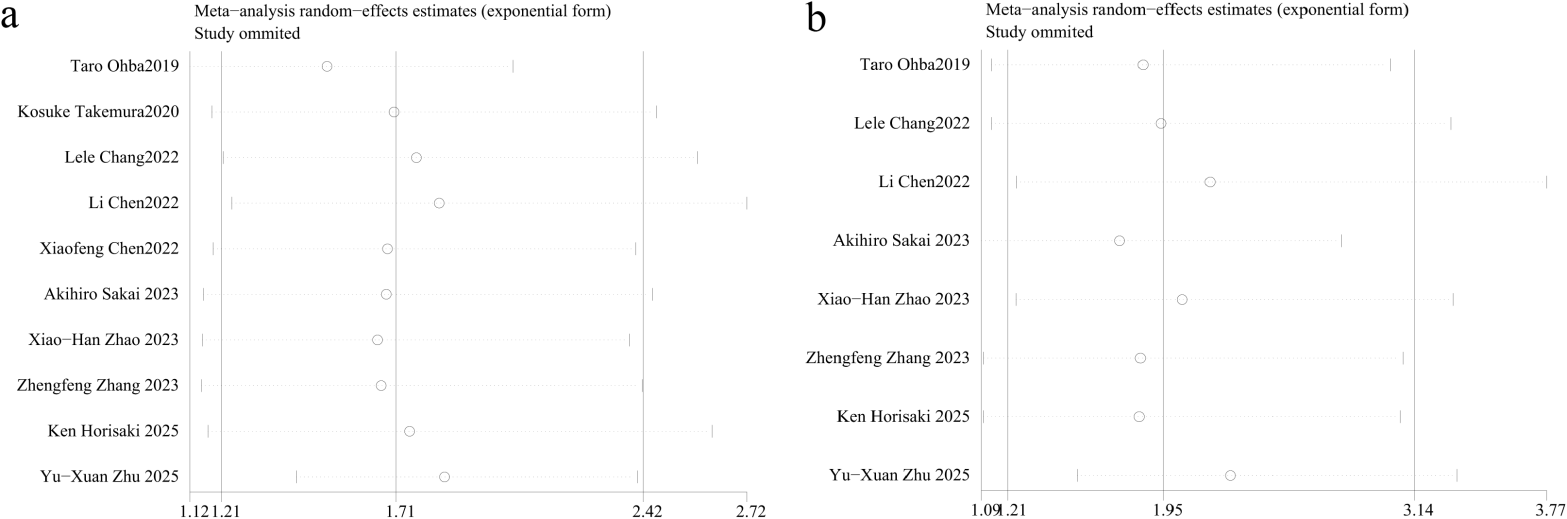
Sensitivity analysis of CONUT on progressive-free survival **(a)** and overall survival **(b)** in patients treated with immune checkpoint inhibitors.

Sensitivity analysis showed that the combined effect size was consistent but the heterogeneity was not eliminated after sensitivity analysis (**Figure 5**).

## Discussion

This study systematically explored the prognostic value of the CONUT score in cancer patients treated with ICIs. The key finding was that a higher baseline CONUT score was significantly associated with poorer treatment responses (ORR and DCR), as well as shorter survival (PFS and OS). The results corroborate the central position of the “nutrition - inflammation - immunity” axis in cancer immunotherapy. Malnutrition can impair T-cell function and promote an immunosuppressive microenvironment. CONUT score is a comprehensive index based on ALB, T-CHOL and TLC, which can reflect immune response, nutritional status and systemic inflammatory response. Therefore, it emerges as a potential and readily accessible biomarker for predicting the outcomes of ICIs.

Previous studies have demonstrated the value of CONUT in surgery therapy (51, 52), chemotherapy (38), radiotherapy (53, 54), and targeted therapy (55), and have shown a good prognostic effect. The results of this study also suggest that it will continue to shine in the field of immunotherapy. However, the mechanism of association between high CONUT score and poor prognosis in tumor patients is not clear. As a parameter to evaluate nutritional status and liver synthesis ability, ALB is affected by nutritional status, inflammation, infection and hydration status (56). In the field of immunity, ALB can reflect the aggressive behavior and inflammatory state of tumors. Inflammatory cytokines such as interleukin-6 secreted by the tumor microenvironment may contribute to ALB reduction (57). Hypoproteinemia can lead to physiological dysfunction, including loss of drug efficacy, abnormal activation of systemic inflammation, and impaired immune function (58), and hypoproteinemia often predicts a poor prognosis for cancer patients (59–61). Cholesterol can affect antioxidant reserve and inflammatory response, and is related to tumor load and nutritional status (62). Cholesterol in biofilms can affect the plasma membrane environment and effector function of a variety of immune cells, such as T cells, NK cells and neutrophils (63, 64). It was found that cholesterol can reduce endoplasmic reticulum, leading to endoplasmic reticulum stress and promoting the expression of immunosuppressive genes (65). Cholesterol also involved in signal transduction of various receptors and formation of cellular immune synapses, thus affecting the effect of immunotherapy (66–68). Hypocholesterolemia was reported to be associated with worse clinical survival in cancers, including hematologic malignancies, lung cancer, and renal cell carcinoma (69–71). Lymphocyte is one of the key factors in immune monitoring and editing, and plays a crucial role in cell-mediated immune system. TLC can reflect immune responses and systemic inflammatory responses (72), and may serve as a surrogate marker in hosting immunological incompetence (73). Weakened immune monitoring caused by low TLC can provide favorable conditions for tumor cell growth and progression. Increasing evidence has shown that low TLC leads to poor survival in cancer patients (74, 75). These three components were not isolated but were tightly coupled through the “nutrition-inflammation-immunity” axis. They synergistically shape the tumor immune microenvironment by collectively impairing T cell function, driving macrophage polarization toward the M2 phenotype, and fostering an immunosuppressive cytokine environment. Systemic inflammation and malnutrition lead to the depletion of immune resources and metabolic reprogramming, while abnormal cholesterol metabolism, in turn, solidifies the immunosuppressive state by inhibiting T cells and promoting M2 polarization, further exacerbating inflammation and metabolic exhaustion. Therefore, CONUT, as an immunonutritional index, may be a good predictor of clinical efficacy of immunotherapy.

However, the most thought-provoking finding of this study is not its overall predictive power, but the significant heterogeneity behind it. CONUT score has been proven to be a be a robust prognostic indicator independent of clinical factors, and no significant associations were found in terms of patients’ gender, cancer stage, and cancer type (76). Through subgroup analysis, we have revealed that the relationship between the CONUT score and the efficacy of immunotherapy is not a simple linear association, but is complexly regulated by multiple factors. The cut-off value of CONUT score might be the key to heterogeneity and the core in determining its predictive efficacy. The cut-value of CONUT at 2 has been proven to be the optimal critical value for predicting the survival rate of patients with gastric cancer who receive radical treatment (77), while another study found that the optimal critical value for predicting the 5-year survival rate of gastric cancer patients is 3 (78). However, the cut-value of the CONUT score at 1 can significantly predict the disease-free survival and OS of breast cancer patients after surgery (79). These research results indicate that for different types of tumors, treatment methods, and prognostic indicators, the most suitable cut-off value for CONUT varies as well. Our subgroup analysis results clearly depict a “dose-effect” relationship. When overly lenient cut-off value (such as 2 points) is adopted, the discriminatory ability of the CONUT score for PFS and OS vanishes. As the cut-off value increases to 3 points or 4 points, its predictive efficacy for PFS and OS gradually emerges and stabilizes. Previous study has also noted that the ability of CONUT to predict the survival of patients with hepatocellular carcinoma becomes insignificant for lower cut-off values (2, 3), but it remains significant for higher cut-off values (4, 5) (80). This indicates that grouping patients with only a low nutritional risk (score of 2-4) together with those with normal nutrition will significantly dilute the prognostic predictive power of the CONUT score. It is likely due to the fact that a CONUT score within the range of 2–3 may indicate mild nutritional depletion. Under active clinical supportive treatment, this condition may be partially reversed. Whereas, when the score reaches or exceeds 3, it indicates moderate to severe nutritional and immune exhaustion. Our subgroup analysis also revealed that when the cut-off value was 3, the predictive capabilities of PFS and OS were the most robust and stable, with the least heterogeneity. This is consistent with the previous results of the meta-analysis, that is, the CONUT score with cut-off value of 3 before treatment shows a correlation with the clinical outcomes of various malignant tumors (81). Numerous studies have indicated that when CONUT effectively predicts the PFS or OS of patients with colorectal cancer, epithelial ovarian cancer, thyroid cancer, bladder cancer, and breast cancer, the optimal cut-off value is uniformly 3 (55, 82–85). At this point, the physiological damage caused by the nutritional and immune depletion to the body is already severe enough to directly translate into faster disease progression and a shorter survival period. Regarding the inconsistency in OS at the cut-off values of 3 and 4, on the one hand, this may be due to the influence of sample size and the number of subsequent treatment lines; on the other hand, it indicates that the impact of nutritional status on the direct progression of tumors is more direct and sensitive than its impact on the overall survival of patients.

Compared with low CONUT group, patients with urothelial cancer and hepatocellular carcinoma in high CONUT group had poorer OS, and subgroup analysis based on country (Japan, China) yielded similar results (80, 86). Another study has also shown that based on the subgroup analysis by country (Turkey, China, Japan) and the cut-off value (4–6), the effectiveness of CONUT in predicting the OS of patients with hematological malignancies remains unchanged (87). We observed that the differences among countries constitute another dimension of heterogeneity. In the Japanese population, the consistent negative prognostic effect of a high CONUT score on PFS and OS was strongly confirmed. However, in the Chinese population, its prognostic effect on OS was uncertain. These cross-regional differences may stem from various factors, including the genetic background of the population, physical differences, tumor epidemiological characteristics, as well as subtle variations in clinical practice, such as the choice of anti-tumor treatment regimens, the intensity of nutritional support therapy, and even differences in the healthcare system. This warns us that when applying the CONUT score, which is a tool derived from a specific population, across borders, we must consider its regional applicability and conduct necessary local verification.

In the subgroup analysis, some heterogeneity still persisted, indicating that unmeasured confounding factors might influence the results, such as the vast diversity in cancer types, variations in ICI drugs and treatment lines, and differences in combination regimens. Nevertheless, we were still able to examine the effects of important confounding factors such as country and cut-off values on the magnitude of the effect. Therefore, future research urgently needs to explore and verify the optimal critical values for specific immunotherapy populations. If treatment response and survival can be predicted before treatment with ICIs, the prognosis may be improved by giving supportive treatment early, thereby saving treatment resources. CONUT score may be a more appropriate predictor. It reflects not only nutritional status but also systemic inflammation and immune responses, potentially providing a more balanced assessment than other measures. It is simple, economical, and non-invasive because it is calculated by analyzing routine biochemical parameters at no additional cost to the patient.

The present study has some limitations. Firstly, all included studies were retrospective, limiting causal inference and introducing risk of selection or confounding bias. Secondly, this study only included 10 studies, resulting in a relatively low test efficacy. Thirdly, the total sample size and number of studies were modest, limiting powerful subgroup analyses by cancer type or treatment regimen. Finally, although CONUT was convenient, it may be influenced by factors such as infection, liver dysfunction, corticosteroid use, or timing of measurement relative to ICI initiation, which could act as confounders.

## Conclusions

This meta-analysis confirms the potential value of the CONUT score as a predictive tool for the efficacy and prognosis of ICIs. Malnutrition and inflammatory status, as intervenable host factors, are closely linked to outcomes of immunotherapy through the CONUT score. Our research goes beyond merely validating the correlation. Through in-depth heterogeneity analysis, it paves the way for the future development of this field. Future research should shift the focus from “whether it is effective” to “how to apply it optimally”. Large-scale prospective cohort studies should be conducted to determine the optimal cutoff values for specific cancer types that receive specific ICI regimens. Additionally, randomized controlled trials should be carried out to compare the effects of standard treatment versus enhanced nutritional/anti-inflammatory support based on the CONUT on the efficacy of ICI. This would elevate the CONUT from a passive prognostic predictor to a dynamic management tool capable of guiding clinical decision-making and actively improving patients’ survival.

## Data Availability

The original contributions presented in the study are included in the article/**Supplementary Material**. Further inquiries can be directed to the corresponding authors.
