# Supplementary material for: Controlling nutritional status score predicts clinical outcome in cancer patients treated with immune checkpoint inhibitor: a systematic review and meta-analysis
Source: Front Immunol. 2026 Feb 23;17:1751492. doi: 10.3389/fimmu.2026.1751492 (PMC12967986; doi:10.3389/fimmu.2026.1751492)
Supplement: Supplementary file 1 [file DataSheet1.docx]

**PubMed (NLM): 12 results**

#1 "Neoplasms"[Mesh] OR Tumor [Title/Abstract] OR Neoplasm [Title/Abstract] OR Tumors [Title/Abstract] OR Neoplasia [Title/Abstract] OR Neoplasias [Title/Abstract] OR Cancer [Title/Abstract] OR Cancers [Title/Abstract] OR Malignant Neoplasm [Title/Abstract] OR Malignancy [Title/Abstract] OR Malignancies [Title/Abstract] OR Malignant Neoplasms [Title/Abstract] OR Neoplasm, Malignant [Title/Abstract] OR Neoplasms, Malignant [Title/Abstract] OR Benign Neoplasms [Title/Abstract] OR Benign Neoplasm [Title/Abstract] OR Neoplasms, Benign [Title/Abstract] OR Neoplasm, Benign [Title/Abstract]

#2 "Immune Checkpoint Inhibitors"[Mesh] OR Checkpoint Inhibitors, Immune [Title/Abstract] OR Immune Checkpoint Inhibitor [Title/Abstract] OR Checkpoint Inhibitor, Immune [Title/Abstract] OR Immune Checkpoint Blockers [Title/Abstract] OR Checkpoint Blockers, Immune [Title/Abstract] OR Immune Checkpoint Blockade [Title/Abstract] OR Checkpoint Blockade, Immune [Title/Abstract] OR Immune Checkpoint Inhibition [Title/Abstract] OR Checkpoint Inhibition, Immune [Title/Abstract] OR PD-L1 Inhibitors [Title/Abstract] OR PD L1 Inhibitors [Title/Abstract] OR PD-L1 Inhibitor [Title/Abstract] OR PD L1 Inhibitor [Title/Abstract] OR Programmed Death-Ligand 1 Inhibitors [Title/Abstract] OR Programmed Death Ligand 1 Inhibitors [Title/Abstract] OR PD-1-PD-L1 Blockade [Title/Abstract] OR Blockade, PD-1-PD-L1 [Title/Abstract] OR PD 1 PD L1 Blockade [Title/Abstract] OR CTLA-4 Inhibitors [Title/Abstract] OR CTLA 4 Inhibitors [Title/Abstract] OR CTLA-4 Inhibitor [Title/Abstract] OR CTLA 4 Inhibitor [Title/Abstract] OR Cytotoxic T-Lymphocyte-Associated Protein 4 Inhibitors [Title/Abstract] OR Cytotoxic T Lymphocyte Associated Protein 4 Inhibitors [Title/Abstract] OR Cytotoxic T-Lymphocyte-Associated Protein 4 Inhibitor [Title/Abstract] OR Cytotoxic T Lymphocyte Associated Protein 4 Inhibitor [Title/Abstract] OR PD-1 Inhibitors [Title/Abstract] OR PD 1 Inhibitors [Title/Abstract] OR PD-1 Inhibitor [Title/Abstract] OR Inhibitor, PD-1 [Title/Abstract] OR PD 1 Inhibitor [Title/Abstract] OR Programmed Cell Death Protein 1 Inhibitor [Title/Abstract] OR Programmed Cell Death Protein 1 Inhibitors [Title/Abstract]

#3 Controlling Nutritional Status [Title/Abstract] OR CONUT [Title/Abstract]

#4 English [Language]

#5 #1 AND #2 AND #3 AND #4

**Web of science (Clarivate): 45 results**

#1 TS=(Tumor) OR TS=(Neoplasm) OR TS=(Tumors) OR TS=(Neoplasia) OR TS=(Neoplasias) OR TS=(Cancer) OR TS=(Cancers) OR TS=(Malignant Neoplasm) OR TS=(Malignancy) OR TS=(Malignancies) OR TS=(Malignant Neoplasms) OR TS=(Neoplasm, Malignant) OR TS=(Neoplasms, Malignant) OR TS=(Benign Neoplasms) OR TS=(Benign Neoplasm) OR TS=(Neoplasms, Benign) OR TS=(Neoplasm, Benign)

#2 TS=(Checkpoint Inhibitors, Immune) OR TS=(Immune Checkpoint Inhibitor) OR TS=(Checkpoint Inhibitor, Immune) OR TS=(Immune Checkpoint Blockers) OR TS=(Checkpoint Blockers, Immune) OR TS=(Immune Checkpoint Blockade) OR TS=(Checkpoint Blockade, Immune) OR TS=(Immune Checkpoint Inhibition) OR TS=(Checkpoint Inhibition, Immune) OR TS=(PD-L1 Inhibitors) OR TS=(PD L1 Inhibitors) OR TS=(PD-L1 Inhibitor) OR TS=(PD L1 Inhibitor) OR TS=(Programmed Death-Ligand 1 Inhibitors) OR TS=(Programmed Death Ligand 1 Inhibitors) OR TS=(PD-1-PD-L1 Blockade) OR TS=(Blockade, PD-1-PD-L1) OR TS=(PD 1 PD L1 Blockade) OR TS=(CTLA-4 Inhibitors) OR TS=(CTLA 4 Inhibitors) OR TS=(CTLA-4 Inhibitor) OR TS=(CTLA 4 Inhibitor) OR TS=(Cytotoxic T-Lymphocyte-Associated Protein 4 Inhibitors) OR TS=(Cytotoxic T Lymphocyte Associated Protein 4 Inhibitors) OR TS=(Cytotoxic T-Lymphocyte-Associated Protein 4 Inhibitor) OR TS=(Cytotoxic T Lymphocyte Associated Protein 4 Inhibitor) OR TS=(PD-1 Inhibitors) OR TS=(PD 1 Inhibitors) OR TS=(PD-1 Inhibitor) OR TS=(Inhibitor, PD-1) OR TS=(PD 1 Inhibitor) OR TS=(Programmed Cell Death Protein 1 Inhibitor) OR TS=(Programmed Cell Death Protein 1 Inhibitors)

#3 TS=(Controlling Nutritional Status) OR TS=(CONUT)

#4 LA=(English)

#5 #1 AND #2 AND #3 AND #4

**MEDLINE (Clarivate): 46 results**

#1 TS=(Tumor) OR TS=(Neoplasm) OR TS=(Tumors) OR TS=(Neoplasia) OR TS=(Neoplasias) OR TS=(Cancer) OR TS=(Cancers) OR TS=(Malignant Neoplasm) OR TS=(Malignancy) OR TS=(Malignancies) OR TS=(Malignant Neoplasms) OR TS=(Neoplasm, Malignant) OR TS=(Neoplasms, Malignant) OR TS=(Benign Neoplasms) OR TS=(Benign Neoplasm) OR TS=(Neoplasms, Benign) OR TS=(Neoplasm, Benign)

#2 TS=(Checkpoint Inhibitors, Immune) OR TS=(Immune Checkpoint Inhibitor) OR TS=(Checkpoint Inhibitor, Immune) OR TS=(Immune Checkpoint Blockers) OR TS=(Checkpoint Blockers, Immune) OR TS=(Immune Checkpoint Blockade) OR TS=(Checkpoint Blockade, Immune) OR TS=(Immune Checkpoint Inhibition) OR TS=(Checkpoint Inhibition, Immune) OR TS=(PD-L1 Inhibitors) OR TS=(PD L1 Inhibitors) OR TS=(PD-L1 Inhibitor) OR TS=(PD L1 Inhibitor) OR TS=(Programmed Death-Ligand 1 Inhibitors) OR TS=(Programmed Death Ligand 1 Inhibitors) OR TS=(PD-1-PD-L1 Blockade) OR TS=(Blockade, PD-1-PD-L1) OR TS=(PD 1 PD L1 Blockade) OR TS=(CTLA-4 Inhibitors) OR TS=(CTLA 4 Inhibitors) OR TS=(CTLA-4 Inhibitor) OR TS=(CTLA 4 Inhibitor) OR TS=(Cytotoxic T-Lymphocyte-Associated Protein 4 Inhibitors) OR TS=(Cytotoxic T Lymphocyte Associated Protein 4 Inhibitors) OR TS=(Cytotoxic T-Lymphocyte-Associated Protein 4 Inhibitor) OR TS=(Cytotoxic T Lymphocyte Associated Protein 4 Inhibitor) OR TS=(PD-1 Inhibitors) OR TS=(PD 1 Inhibitors) OR TS=(PD-1 Inhibitor) OR TS=(Inhibitor, PD-1) OR TS=(PD 1 Inhibitor) OR TS=(Programmed Cell Death Protein 1 Inhibitor) OR TS=(Programmed Cell Death Protein 1 Inhibitors)

#3 TS=(Controlling Nutritional Status) OR TS=(CONUT)

#4 LA=(English)

#5 #1 AND #2 AND #3 AND #4

**Embase (Elsevier platforms): 28 results**

#1 'malignant neoplasm'/exp OR 'cancer':ti,ab OR 'cancers':ti,ab OR 'malignant neoplasia':ti,ab OR 'malignant neoplastic disease':ti,ab OR 'malignant tumor':ti,ab OR 'malignant tumour':ti,ab OR 'neoplasia':ti,ab OR 'malignant':ti,ab OR 'tumor, malignant':ti,ab OR 'tumour, malignant':ti,ab

#2 'immune checkpoint inhibitor'/exp OR 'immune checkpoint inhibitor':ti,ab OR 'immune checkpoint inhibitors':ti,ab

#3 'controlling nutritional status':ti,ab OR 'CONUT':ti,ab

#4 #1 AND #2 AND #3AND [english]/lim
