# Supplementary material for: Controlling nutritional status score predicts clinical outcome in cancer patients treated with immune checkpoint inhibitor: a systematic review and meta-analysis
Source: Front Immunol. 2026 Feb 23;17:1751492. doi: 10.3389/fimmu.2026.1751492 (PMC12967986; doi:10.3389/fimmu.2026.1751492)
Supplement: Supplementary file 2 [file Table1.docx]

Supplementary Table 1 The outcome measures of included studies

| Study | PFS | OS | ORR, % | DCR, % |
| --- | --- | --- | --- | --- |
| Taro Ohba 2019 | 5.88 (2.13-16.67) | 4.00 (1.02-20.00) | 50% vs. 30% | 84.6% vs. 50% |
| Kosuke Takemura 2020 | 1.91 (0.65-5.61) | NR | NR | NR |
| Lele Chang 2022 | 1.30 (0.52–3.24) | 2.06 (1.03–4.10) | 57.14% vs. 37.04% | 80.95% vs. 51.85% |
| Li Chen 2022 | 1.12 (0.60–2.08) | 1.04 (0.56–1.94) | NR | NR |
| Xiaofeng Chen 2022 | 3.57 (0.36-33.33) | NR | NR | NR |
| Akihiro Sakai 2023 | 2.00 (1.05-3.82) | 4.03 (1.94-8.33) | 67.65% vs. 82.35% | 85.29% vs. 88.24% |
| Xiao-Han Zhao 2023 | 2.36 (1.01-5.48) | 0.52 (0.05-5.88) | NR | NR |
| Zhengfeng Zhang 2023 | 2.08 (1.14-3.85) | 2.70 (1.59-4.76) | NR | NR |
| Ken Horisaki 2025 | 1.65 (1.06–2.57) | 2.68 (1.67–4.30) | 29.85% vs. 12.5% | 64.18% vs. 25% |
| Yu-Xuan Zhu 2025 | 0.95 (0.79–1.15) | 1.04 (0.86–1.24) | NR | NR |

PFS: progression-free survival; OS: overall survival; ORR: objective response rate; DCR: disease control rate; NR: not reported
