# Supplementary material for: Controlling nutritional status score predicts clinical outcome in cancer patients treated with immune checkpoint inhibitor: a systematic review and meta-analysis
Source: Front Immunol. 2026 Feb 23;17:1751492. doi: 10.3389/fimmu.2026.1751492 (PMC12967986; doi:10.3389/fimmu.2026.1751492)
Supplement: Supplementary file 3 [file Table2.docx]

Supplementary Table 2 Quality assessment of included studies using Newcastle Ottawa Scale

| Study | Selection | Comparability | Outcome | Total points |
| --- | --- | --- | --- | --- |
|  | (1) Representativeness of exposed cohort  (2) Selection of non-exposed cohort  (3) Ascertainment of exposure  (4) Demonstration that the outcome of interest was not present at the start of the study (☆☆☆☆) | Comparability of cohorts on the basis of the design or analysis (☆☆) | (1) Assessment of outcome  (2) Was follow-up long enough for outcomes to occur  (3) Adequacy of follow-up of cohorts (☆☆☆) | 9 |
| Taro Ohba2019 | ☆1), ☆2), ☆3), ☆4) | ☆ | ☆1), ☆2), ☆3) | 8 |
| Kosuke Takemura2020 | ☆1), ☆2), ☆3), ☆4) | ☆ | ☆1), ☆2), ☆3) | 8 |
| Lele Chang2022 | ☆1), ☆2), ☆3), ☆4) | ☆ | ☆1), ☆2), ☆3) | 8 |
| Li Chen2022 | ☆1), ☆2), ☆3), ☆4) | ☆ | ☆1), ☆2), ☆3) | 8 |
| Xiaofeng Chen2022 | ☆1), ☆2), ☆3), ☆4) | ☆ | ☆1), ☆2), ☆3) | 8 |
| Akihiro Sakai 2023 | ☆1), ☆2), ☆3), ☆4) | ☆ | ☆1), ☆2), ☆3) | 8 |
| Xiao-Han Zhao 2023 | ☆1), ☆2), ☆3), ☆4) | ☆ | ☆1), ☆2), ☆3) | 8 |
| Zhengfeng Zhang 2023 | ☆1), ☆2), ☆3), ☆4) | ☆ | ☆1), ☆2), ☆3) | 8 |
| Ken Horisaki 2025 | ☆1), ☆2), ☆3), ☆4) | ☆ | ☆1), ☆2), ☆3) | 8 |
| Yu-Xuan Zhu 2025 | ☆1), ☆2), ☆3), ☆4) | ☆☆ | ☆1), ☆2), ☆3) | 9 |
